# Supplementary material for: WordCommentsAnalyzer: A windows software tool for qualitative research
Source: F1000Res. 2018 Sep 4;7:536. Originally published 2018 May 3. [Version 2] doi: 10.12688/f1000research.14819.2 (PMC6137411; doi:10.12688/f1000research.14819.2)
Supplement: Supplementary file 3 [file f1000research-7-17614-s0002.tgz › b2a79496-4ada-47c4-9682-a99ce867836d.zip!/Eight Iranian Health Journals/Salmand_Iranian Journal of Ageing _18.docx]

© Salmand_Iranian Journal of Ageing . This work is licensed under CC BY-NC 4.0 (https://creativecommons.org/licenses/by-nc/4.0/).

Volume & Issue: دوره 8 - شماره 4

Title: بررسی رابطه بین پلی‌فارماسی و عوامل اجتماعی جمعیت‌شناختی در سالمندان آسایشگاه خیریه کهریزک در سال ۱۳۸۹

http://salmandj.uswr.ac.ir/article-1-523-fa.html

اهداف: بررسی ارتباط بین پلی‌فارماسی و عوامل اجتماعی جمعیت‌شناختی شامل سن، جنسیت، تحصیلات و سیگار در سالمندان ساکن آسایشگاه خیریۀ کهریزک مواد و روش‌ها: درطول مطالعه‌ای توصیفی تحلیلی مقطعی، ۲۳۷ سالمند ۶۰‌ساله و بالاتر (۱۳۴ نفر زن) ساکن آسایشگاه خیریۀ کهریزک، به‌روش تصادفی خوشه‌ای انتخاب شدند. سپس، ازنظر تعداد داروهای مصرفی و ارتباط پلی‌فارماسی (مصرف هم‌زمان پنج دارو یا بیشتر) با عوامل اجتماعی جمعیت‌شناختی بررسی شدند. یافته‌ها: میانگین تعداد داروهای مصرفی (۳/۲۲±) به‌ازای هر مددجو، ۵/۱۰ بود. فراوانی پلی فارماسی، ۵۲/۳درصد بود. درمقایسه‌ با گروه سِنی ۶۰ تا ۶۹ سال، شانس پلی‌فارماسی در گروه سِنی ۷۰ تا ۷۹  سال افزایش یافت (OR=1/09, 95%CI: 2/25-0/53) و در گروه سِنی ۸۰ساله به‌بالا کاهش پیدا کرد (OR=0/68, 95%CI: 1/24-0/37) که ازنظر آماری معنادار نبود. فراوانی و شانس پلی‌فارماسی در مردان کمتر از زنان بود (OR=0/68, 95%CI: 1/24-0/37)؛ ولی این رابطه ازنظر آماری معنادار نبود. شیوع و شانس پلی‌فارماسی به‌طور معناداری، در سیگاری‌ها کمتر بود (OR=0/36, 95%CI: 0/79-0/17). براین‌اساس، رابطۀ مستقیم و معنادار بین افزایش سال‌های تحصیلات رسمی و پلی‌فارماسی وجود داشت (OR=1/09, 95%CI: 1/18-1/01). نتیجه‌گیری: میانگین تعداد دارو‌های مصرفی و شیوع پلی‌فارماسی در آسایشگاه کهریزک بالا بود. افزایش سال‌‌های تحصیلات رسمی و سیگارنکشیدن، ریسک‌فاکتور‌های پلی‌فارماسی در این جمعیت بود. درکل، رابطۀ معناداری بین سن و جنسیت با پلی‌فارماسی مشاهده نشد.

Title: پیش‌بینی و پایش زمین‌خوردگی سالمندان بر اساس مدل‌سازی الگوهای حرکتی دریافت‌شده از تصاویر ویدیویی

http://salmandj.uswr.ac.ir/article-1-581-fa.html

اهداف: با افزایش جمعیت سالمندان در اغلب کشورهای جهان، طراحی سیستمی قابل اعتماد و هوشمند جهت پایش و نظارت وضعیت جسمانی آنان ضروری به نظر می‌رسد. سالیانه بر اثر زمین‌خوردگی، هزاران نفر از سالمندان با صدمات جبران‌ناپذیری همچون شکستگی مفاصل، استخوان‌ها و دیگر اعضای بدن خود و حتی در مواردی با مرگ مواجه می‌شوند. مواد و روش‌ها: در این مقاله بر مبنای تحلیل تصاویر اخذ شده از حرکت سالمندان، سیستمی کارآمد طراحی شده که در فاز نخست، راه رفتن غیر طبیعی سالمندان را تشخیص می‌دهد و در گام بعدی نحوه حرکت آن‌ها را مدل‌سازی می‌کند. سپس با ترکیب چندین ویژگی با اهمیت نظیر برآورد زاویه بدن شخص نسبت به افق، ارزیابی کمیت‌های حرکتی، حافظه حرکت، راستای حرکت و سرعت فرد، زمین خوردگی احتمالی شناسایی می‌شود. پیاده‌سازی سیستم بر مجموعه‌ای شامل 57425 فریم تصویری دریافت‌ شده از مرکز نگهداری سالمندان فرزانگان شهر مشهد و رشته‌های ویدئویی دربردارنده وقایع زمین‌خوردگی صورت پذیرفت. تمامی رشته‌ها به صورت تصادفی به 4 دسته Movie با فرمت AVI با رزولوشن 120×160 پیکسل با نرخ 15 فریم بر ثانیه تبدیل شدند. یافته‌ها: نتایج شبیه‌سازی الگوریتم، نشان دهنده عملکرد مطلوب سیستم و دقت مناسب آن در شناسایی حوادث زمین خوردگی است. دقت متوسط (AAC)، نرخ آشکار سازی (DR) و نرخ هشدار اشتباه (FAR) سیستم پیشنهادی در سطح قابل قبولی به ترتیب %94، %91/92 و %52/5 است. حساسیت %92 و ویژگی% 47/94، نشان‌دهنده توانایی مطلوب سیستم در جداسازی وقایع شبیه به زمین خوردگی و زمین خوردگی‌های واقعی است. نتیجه‌گیری: مزیت‌های ویژه‌ای چون سرعت بالای الگوریتم در پردازش داده‌ها و ارائه دقت و حساسیت مناسب در عملکرد سبب شده تا سیستم پیشنهادی از سایر تکنیک‌های مشابه متمایز شود و از این رو به کارگیری آن در مراکز مختلف نگهداری سالمندان و منازل مسکونی، نظارت هوشمند و بلادرنگ افراد را در زمان زمین‌خوردگی به دنبال خواهد داشت.

Title: مقایسه ترس از مرگ در سالمندان ساکن منازل با سالمندان ساکن سرای سالمندان شهر اصفهان

http://salmandj.uswr.ac.ir/article-1-543-fa.html

اهداف: ازطرفی، افزایش روزافزون جمعیت سالمندان و ازطرف‌ِدیگر، تغییرات اجتماعی‌‌اقتصادی و شیوۀ زندگی فردی و خانوادگی باعث افزایش تعداد مؤسسات نگهداری سالمندان شده است. هدف پژوهش حاضر، مقایسۀ ترس از مرگ در بین سالمندان مقیم در سرای سالمندان و ساکن در منازل شهر اصفهان است. مواد و روش‌ها: این مطالعه از نوع مقطعی‌تحلیلی و جامعۀ آماری موضوع مطالعه، تمام سالمندان ۶۰ سال به‌بالای ساکن در مناطق چهارده‌گانۀ شهر اصفهان و سالمندان مقیم در سرای سالمندان صادقیه است. شرکت‌کنندگان در این پژوهش، ۳۰۰ سالمند بودند که ۱۰۲ نفر مقیم سرای سالمندان، یعنی ۳۱ مرد و ۷۱ زن و ۱۹۸ نفر مقیم جامعه بودند؛ یعنی ۹۷ مرد و ۱۰۱ زن. سالمندان مقیم جامعه، با استفاده از روش نمونه‌گیری خوشه‌ای و سالمندان مقیم سرای سالمندان به شیوۀ نمونه‌گیری در دسترس انتخاب شدند و ازطریق پرسش‌نامۀ ترس از مرگ کالتلستر در معرض ارزیابی قرار گرفتند. برای تجزیه‌وتحلیل اطلاعات گردآوری‌شده، از روش‌های آماری پارامتریک آزمون کای اسکوئر و تی مستقل استفاده شده است. یافته‌ها: یافته‌های این پژوهش نشان داد میانگین نمرۀ کل ترس از مرگ نیز در گروه سالمندان ساکن منازل ۱۵/۶±۷۰/۵ و به‌طور معناداری، بیشتر از گروه سالمندان ساکن سرای سالمندان بود (۰/۰۰۱=P). نتیجه‌گیری: نتایج این مطالعه، نشان داد ترس از مرگ در سالمندان ساکن در منازل شهر اصفهان، بیشتر از سالمندان ساکن در سرای سالمندان است. لازم است دست‌اندرکاران نظام‌های بهداشتی در تمامی بخش‌ها، این نکته را مدنظر قرار دهند و با ارزیابی علل و استمداد از سایر ارگان‌های مربوط، گام مؤثری برای کاهش ترس از مرگ سالمندان بردارند.

Title: بررسی شیوع ناتوانی و عوامل خطر آن در سالمندان شهر اصفهان در سال ۱۳۹۰

http://salmandj.uswr.ac.ir/article-1-508-fa.html

اهداف: باتوجه‌به افزایش جمعیت سالمندان در کشور، این مطالعه با هدف تعیین میزان ناتوانی سالمندان و عوامل مرتبط با آن، به‌منظور برنامه‌ریزی برای پیشگیری از ناتوانی و کاهش عوارض آن در افراد سالمند درنظر گرفته شد. مواد و روش‌ها: این پژوهش به روش مقطعی و توصیفی‌تحلیلی بر روی ۳۰۰ نفر از سالمندان ۶۰ سال و بیشتر از ۶۰ سال شهر اصفهان، به روش نمونه‌گیری خوشه‌ای، در هشت منطقه از مناطق چهارده‌گانۀ شهر انجام شد. روش گردآوری داده‌ها ازطریق مراجعه به در منازل افراد سالمند و مصاحبه با آنان بوده است. ابزار جمع‌آوری اطلاعات، پرسش‌نامه‌ای مشتمل بر دو بخش بود که بخش اول دربارۀ مشخصات دموگرافیکی، اقتصادی‌اجتماعی، ابتلا به بیماری‌ها و نیاز‌های سالمندان تحت مطالعه و بخش دوم، پرسش‌نامۀ استاندارد WHODASI از منابع سازمان بهداشت جهانی بود. داده‌ها با استفاده از آمار توصیفی و تحلیلی تجزیه‌وتحلیل شدند. یافته‌ها: میانگین سِنی کل افراد موضوع مطالعه، ۷۱/۴ سال و میانه و نمای سن آنان ۷۰ سال بود. در میان سالمندان تحتِ مطالعه، ۱۵/۷درصد بدون ناتوانی بودند و بیشتر آن‌ها، یعنی ۳۳/۳درصد، میزان ناتوانی‌شان کم بود. درمقابل، بیشترین میزان بدون ناتوانی، یعنی ۶۳/۳درصد، بدون درنظرگرفتن افراد شاغل، مربوط به حیطۀ سازش و زندگی با مردم بود. این درحالی است که بیشترین میزان ناتوانی خیلی شدید در حیطۀ تحرک و جابه‌جایی، ۳۲/۷درصد و پس از آن، در حیطۀ وظایف روزمره، ۲۵/۳درصد دیده شد. با افزایش سن، نمرۀ ناتوانی زیاد (۰/۴=r) شد و با افزایش درآمد، نمرۀ ناتوانی (۰/۳-=r) کاهش معنادار نشان داد. همچنین، زنان، بیوه‌ها، کسانی که با سایر افراد غیر از خانوادۀ خود زندگی می‌کردند، بی‌سوادان و کارگران، به‌طور معناداری ناتوانی بیشتری داشتند. ازنظر عوامل اختلالات و بیماری‌ها، شدت ناتوانی در این دسته از بیماری‌های زیر بود: بیماری‌‌های اسکلتی عضلانی، بی‌اختیاری ادرار و مدفوع، اختلال تکلم، سکتۀ مغزی، نداشتن تعادل، آلزایمر، پارکینسون و اختلالات شناختی و حافظه؛ به‌طوری‌که میانگین نمرۀ ناتوانی از ۱۰۰ به‌طور تقریبی، در مبتلایان به بیماری‌‌های عضلانی‌اسکلتی ۲/۷ برابر، آلزایمر ۲/۶ برابر، پارکینسون ۲/۷ برابر، بی‌اختیاری ادرار و مدفوع، اختلال تکلم، سکتۀ مغزی، اختلال شناخت و حافظه و نیز نداشتن تعادل، دو برابر شد. نتیجه‌گیری: باتوجه‌به اینکه سن از عوامل مؤثر بر ناتوانی بود، لازم است تدابیری برای به‌تعویق‌انداختن ناتوانی در سنین بیشتر و نیز ارائۀ خدمات توان‌بخشی برای حفظ استقلال و بهبود عملکرد افراد سالمند، به‌خصوص زنان سالمند صورت گیرد. براساس نتایج این مطالعه، به‌نظر می‌رسد عمده‌ترین بیماری‌هایی که در سالمندان ایجاد ناتوانی می‌کنند، اختلالات عضلانی‌اسکلتی و افسردگی هستند که دراین‌راستا، لازم است امکانات تشخیص زودرس و درمان و خدمات بازتوانی برای سالمندان فراهم شود.

Title: مقایسه حیطه‌‌های روانی مشکل‌ساز برای دیابت سالمندان دیابتی ساکن در سرای سالمندان کهریزک و ساکنان خانه شهر تهران

http://salmandj.uswr.ac.ir/article-1-540-fa.html

اهداف: با‌توجه‌به افزایش میزان دیابت و رشد سالمندی در کشور، هدف این تحقیق، بررسی و مقایسۀ حیطه‌‌های مشکل‌ساز ناشی از دیابت برای سالمندان دیابتی ساکن در سرای سالمندان و سالمندان ساکن در خانه بوده است. مواد و روش‌ها: این مطالعه از نوع توصیفی‌تحلیلی بوده و دربارۀ سالمندان دیابتی ساکن سرای سالمندان و ساکن شهر تهران صورت گرفته است. از هر گروه، ۹۳ نفر را به‌صورت تصادفی انتخاب کردیم و آزمایش را با رضایت آنان انجام دادیم. برای این مطالعه، پرسش‌نامۀ PAID با ۰/۹۴=α و پایایی ۰/۸۸ را استفاده کردیم و با آزمون‌‌های کولموگراف و تی مستقل و آنوا، در نرم‌افزار SPSS، آنالیز آماری را انجام دادیم. یافته‌ها: آزمون در حیطه‌‌های روانی مدیریت دیابت و مشکلات مرتبط با افسردگی، اختلاف معنا‌داری را نشان می‌دهد (۰/۰۱>P). در این دو حیطه، ساکنان خانۀ سالمندان و در هر دو گروه، نمرات مردان وضعیت بهتری را نشان می‌دهد. نتیجه‌گیری: برای افزایش کیفیت زندگی سالمندان دیابتی، باید علاوه‌بر جنبه‌‌های درمانی، سایر جنبه‌‌های مرتبط با بیماری نیز در‌نظر گرفته شود. توجه به مسائل عاطفی ساکنان سرای سالمندان و مشکلات مراقبتی ساکنان خانه از جملۀ این جنبه‌هاست.

Title: مقایسه کیفیت خواب و سلامت عمومی سالمندان ساکن در خانه و سالمندان ساکن در سرای سالمندان

http://salmandj.uswr.ac.ir/article-1-525-fa.html

اهداف: پژوهش دربارۀ مسائل و مشکلات مرتبط با سالمندی به‌منظور ارتقای کیفیت زندگی این قشر از جمعیت، از دغدغه‌‌‌های عمدۀ جهان امروز به‌شمار می‌رود. تحقیق حاضر با هدف مقایسۀ میزان سلامت عمومی و کیفیت خواب سالمندان ساکن در منزل و سالمندان ساکن در سرای سالمندان انجام شد. مواد و روش‌ها: این پژوهش از نوع تحقیقات توصیفی‌تحلیلی است. جامعۀ آماری این تحقیق تمامی زنان و مردان سالمند شصت سال به‌بالای ساکن در سرای سالمندان و ساکن در منزل شخصی در شهر تبریز بوده است. نمونۀ این پژوهش شامل ۱۰۰ سالمند بود؛ یعنی۵۰ مرد و ۵۰ زن که ۵۰ سالمند ساکن در خانه و ۵۰ سالمند ساکن سرای سالمندان بودند که به روش نمونه‌گیریِ دردسترس انتخاب شدند. برای جمع‌آوری داده‌‌‌ها از دو پرسش‌نامۀ سلامت عمومی گلدبرگ (28-GHQ) و کیفیت خواب پیزبورگ (PSQI) استفاده شد و سپس، داده‌‌‌ها با آزمون تحلیل پراکنش چندمتغیره (MANOVA) بررسی شدند. یافته‌ها: نتایج نشان داد که بین سالمندان ساکن در خانه و سالمندان ساکن در سرای سالمندان از دو نظر تفاوت معنا‌داری، در هر دو گروه، وجود دارد (۰/۰۰۱=P): ۱. ازنظر کیفیت خواب و مؤلفه‌‌‌های آن، شامل کیفیت ذهنی خواب، زمان لازم برای به‌خواب‌رفتن، کل مدت خواب، کارایی خواب معمول، اختلالات خواب، مصرف دارو‌‌های خواب‌آور و اختلال عملکرد روزانه؛ ۲. ازنظر سلامت عمومی و مؤلفه‌‌‌های آن، شامل نشانه‌‌‌های جسمانی، اضطراب، اختلال در عملکرد اجتماعی و افسردگی. نتیجه‌گیری: یافته‌‌‌های پژوهش حاکی‌از آن است که سالمندان ساکن سرای سالمندان در‌مقایسه‌ با سالمندان ساکن در خانه، نشانه‌‌‌های اضطراب و افسردگی و نشانه‌‌‌های جسمانی و اختلال کارکرد اجتماعی بیشتری دارند. همچنین، نتایج نشان داد که سالمندان ساکن سرای سالمندان درمقایسه‌با سالمندان ساکن خانه، کیفیت خواب ضعیفی دارند. در آینده، گسترش مؤسسات نگهداری سالمندی اجتناب‌ناپذیر است؛ بنابراین، توجه هرچه‌بیشتر به اوضاع محیط زندگی سالمندان ساکن مؤسسات، ضروری به‌نظر می‌‌رسد.

Title: تکرارپذیری نسبی و مطلق آزمون Timed Up and Go در سالمندان ساکن اجتماع و جوانان سالم

http://salmandj.uswr.ac.ir/article-1-691-fa.html

اهداف: تکرارپذیری نسبی و مطلق از ویژگی‌های سایکومتریک آزمون هستند که مبنای بسیاری از تصمیم‌گیری‌‌های بالینی قرار می‌گیرند. در بسیاری مواقع، فقط تکرارپذیری نسبی مبنای تصمیم‌گیری و توجه است؛ درحالی‌که تکرارپذیری مطلق هم اهمیت بسیاری دارد. مواد و روش‌ها: ۱۱ سالمند ساکن اجتماع در دامنۀ سِنی ۶۵ سال و بیشتر (۳/۵۸±۶۹/۶۴ سال) و ۲۰ جوان سالم ساکن اجتماع در دامنۀ سِنی ۲۰تا۳۵ سال (۴/۱۵±۲۸/۸۰ سال)، با استفاده از نسخ سه‌گانۀ آزمون Timed Up and Go دو بار با فاصلۀ زمانی ۲ تا۵  روز ارزیابی شدند. یافته‌ها: به‌طورِکلی، غیرهمگن‌بودن جمعیت موضوع مطالعه، باعث افزایش ضریب هم‌بستگی درون‌ طبقه‌ای شده و این ضریب در سالمندان، بیش از جوانان بوده و افزودن فعالیت ثانویه، باعث کاهش آن می‌شود. در این تحقیق، شاخص‌‌های تکرارپذیری مطلق با استفاده از منابع داده‌ها و معادلات مختلف به نتایج کم‌وبیش مشابهی منجر شد. درکل، سالمندان در آزمون مجدد، نیازمند تغییر بیشتری درمقایسه‌با جوانان هستند تا تغییرات مشاهده‌شده تغییری واقعی و نه تصادفی قلمداد شود. سهم خطای تصادفی در متغیر‌‌های تحقیق‌شده در سالمندان، اندکی بیش از جوانان بوده و افزودن فعالیت ثانویه باعث افزایش آن می‌شود. به‌نظر می‌رسد غیرهمگن‌بودن جمعیت، باعث تعدیل شاخص‌‌های تکرار‌پذیری مطلق می‌شود. نتیجه‌گیری: نتایج این تحقیق نشان داد محققان و درمانگران هنگام توجه به تکرارپذیری نسبی، باید به نکاتی همچون همگن‌بودن جمعیت موضوع مطالعه توجه کنند. ازطرفی، در تصمیم‌گیری‌‌های بالینی، توجه به تکرارپذیری مطلق در کنار تکرارپذیری نسبی هم لازم است.

Title: اثربخشی آموزش شادکامی بر افزایش امید در سالمندان

http://salmandj.uswr.ac.ir/article-1-542-fa.html

اهداف: پژوهش حاضر با هدف بررسی اثر‌بخشی آموزش شادکامی بر افزایش امید در سالمندان صورت گرفت. مواد و روش‌ها: مطالعۀ حاضر از نوع کاربردی و طر ح نیمه‌آزمایشی پیش‌آزمون و پس‌آزمون با گروه کنترل نابرابر بود. به‌منظور اجرای این مطالعه، با استفاده از روش نمونه‌گیری دردسترس، ۲۴ نفر از سالمندان مقیم خانۀ سالمندان مشهد که موقعیت ورود به گروه نمونه را داشتند، انتخاب شدند و به‌طور مساوی، در دو گروه آزمایش و کنترل قرار گرفتند. در‌نهایت، به‌دلیل افت تعداد آزمودنی‌ها، نتایج ۲۰ نفر از آن‌ها، یعنی ۱۰ نفر از گروه آزمایشی و ۱۰ نفر از گروه کنترل مطالعه شد. به‌منظور گردآوری داده‌ها، از پرسش‌نامۀ امید اشنایدر (۱۹۹۱) با ضریب پایایی ۸۴ درصد استفاده شد. پس از اجرای پیش‌آزمون، آموزش شادکامی به‌صورت ۱۰ جلسۀ گروهی و به‌مدت ۵ هفته و ۲ جلسه در هفته، برای گروه آزمایش انجام شد. برای گروه کنترل، هیچ مداخلۀ درمانی صورت نگرفت و در لیست انتظار ماندند. پس از پایان آموزش، پس‌آزمون برای هر دو گروه تکرار شد و در مرحلۀ پیگیری، یعنی ۴۵ روز پس از اِعمال متغیر مستقل، آزمون امید برای گروه آزمایش تکرار شد. به‌منظور تجزیه‌و‌تحلیل نتایج، روش تحلیل پراکنش و تی هم‌بسته به‌کار رفت. یافته‌ها: معناداریِ رابطۀ بین متغیر وابستۀ امید و متغیر مستقل را نشان داده شد. بررسی میانگین‌‌های تعدیل‌شدۀ متغیر امید در گروه کنترل و آزمایش، جهت رابطه را نشان داد. مقایسۀ نمرات آزمودنی در گروه آزمایش در مرحلۀ پس‌آزمون و مرحلۀ پیگیری، ازطریق آزمون تی هم‌بسته، نشان‌دهندۀ تفاوت‌نداشتن میانگین‌ها در متغیر امید بود. نتیجه‌گیری: آموزش شادکامی، افزایش امید در سالمندان را به‌دنبال دارد و اثر این آموزش بر متغیر امید، در مرحلۀ پیگیری ثبات داشته است. ازاین‌رو، نظر به اهمیت مسئلۀ سالمندی و مسائل روان‌شناختی آن و با‌توجه‌به نتایج این مطالعه و آثار آموزش شادکامی در سالمندان، می‌توان از مجموعۀ مهارت‌ها و توانمندی‌‌های آموختنی آن، برای کمک به حل مشکلات روان‌شناختی و افزایش میزان امید در سالمندان بهره جست.
